# In vitro comparison of human and murine trabecular meshwork cells: implications for glaucoma research

**DOI:** 10.1038/s41598-024-73057-9

**Published:** 2024-09-23

**Authors:** Fridolin Langer, Maximilian Binter, Xiaonan Hu, Karsten Hufendiek, Roland Meister, Jan Tode, Carsten Framme, Heiko Fuchs

**Affiliations:** University Eye Hospital, Hannover Medical School, 30625 Hannover, Germany

**Keywords:** Preclinical research, Translational research, Experimental models of disease

## Abstract

The trabecular meshwork (TM) is crucial for regulating intraocular pressure (IOP), and its dysfunction significantly contributes to glaucoma, a leading cause of vision loss and blindness worldwide. Although rodents are commonly used as animal models in glaucoma research, the applicability of these findings to humans is limited due to the insufficient understanding of murine TM. This study aimed to compare primary human TM (hTM) and murine TM (mTM) cells in vitro to enhance the robustness and translatability of murine glaucoma models. In this in vitro study, we compared primary hTM and mTM cells under simulated physiological and pathological conditions by exposing both cell types to the glucocorticoid dexamethasone (DEX) and Transforming Growth Factor β (TGFB2), both of which are critical in the pathogenesis of several ophthalmological diseases, including glaucoma. Phagocytic properties were assessed using microbeads. Cells were analyzed through immunocytochemistry (ICC) and Western blot (WB) to evaluate the expression of extracellular matrix (ECM) components, such as Fibronectin 1 (FN1) and Collagen IV (COL IV). Filamentous-Actin (F-Act) staining was used to analyze cross-linked actin network (CLAN) formation. Additionally, we evaluated cytoskeletal components, including Vimentin (VIM), Myocilin (MYOC), and Actin-alpha-2 (ACTA2). Our results demonstrated significant similarities between human and murine TM cells in basic morphology, phagocytic properties, and ECM and cytoskeletal component expression under both homeostatic and pathological conditions in vitro. Both human and murine TM cells exhibited epithelial-to-mesenchymal transition (EMT) after exposure to DEX or TGFB2, with comparable CLAN formation observed in both species. However, there were significant differences in FN1 and MYOC induction between human and murine TM cells. Additionally, MYOC expression in hTM cells depended on fibronectin coating. Our study suggests that murine glaucoma models are potentially translatable to human TM. The observed similarities in ECM and cytoskeletal component expression and the comparable EMT response and CLAN formation support the utility of murine models in glaucoma research. The differences in FN1 and MYOC expression between hTM and mTM warrant further investigation due to their potential impact on TM properties. Overall, this study provides valuable insights into the species-specific characteristics of TM and highlights opportunities to refine murine models for better relevance to human glaucoma.

**Supplementary Information:**

The online version contains supplementary material available at 10.1038/s41598-024-73057-9.

## Introduction

Glaucoma is one of the leading causes of permanent vision loss and visual field deficiency worldwide^1^. While many risk factors for glaucoma have been identified, increased intraocular pressure (IOP) is a primary risk factor for ganglion cell death and vision loss^2^. The intraocular pressure is regulated by aqueous humor formation and outflow, ensuring proper eye function and preventing permanent damage. Ultrafiltration, diffusion, and active secretion contribute to forming aqueous humor, with active secretion by ciliary epithelium cells in the ciliary corpus contributing the majority. Aqueous humor production is relatively constant even under high IOP. Thus, the outflow resistance is responsible for changes in intraocular pressure^3,4^. Therefore, because of lacking alternative therapies, all proven treatment strategies rely on lowering the IOP by pharmacological or surgical intervention to prevent disease progression^5^. The outflow pathways can be divided into a conventional (trabecular) and uveoscleral pathway^6^. The majority of the aqueous humor flows through the conventional pathway, consisting of the trabecular meshwork (TM), the juxtacanicular connective tissues, the endothelial lining of the Schlemm’s canal, the collecting channels, and the aqueous veins, draining into the episcleral venous system^7^. Unlike the conventional outflow route, the uveoscleral pathway has no definite course with channels. Aqueous humor circulates through, around, and between tissues such as the supraciliary space, ciliary muscle, suprachoroidal space, choroidal vessels, sclera, and lymphatic vessels, allowing a relatively constant absorption of aqueous humour across the uvea^8^. The generation and regulation of the IOP is mostly depended on the trabecular pathway, where the outflow resistance is localized in the inner wall region containing the juxtacanalicular tissue of the TM as well as the inner wall endothelium of Schlemm’s canal^9^.

The outflow resistance of the TM is regulated by the accumulation and degradation of extracellular matrix (ECM) proteins produced by TM cells. Increased deposition of ECM causes an increase in outflow resistance^10^. Some findings indicate that changes within the cytoskeleton of TM-cells, the so-called cross-linked actin networks (CLANs), also influence outflow resistance^11^. CLANs are 3-dimensional geodesic dome-like structures first discovered in the actin cytoskeleton of spreading non-muscular cells^12,13^. Usually, CLANs are temporary structures that only occur during the cell attachment and spreading phases. TM cells are exceptional in forming CLANs in vitro while undergoing confluence^14^. This specific property is prevalent in the glaucomatous TM^15,16^.

CLAN formation can be induced by treatment with dexamethasone (DEX)^17^ or Transforming Growth Factor β (TGFB2)^18^. Mathematical models and experimental studies suggest that CLANs contribute to TM’s increased stiffness, thereby increasing outflow resistance^19,20^. Primary TM cells from different species have been used in vitro, including humans^21^, primates^22^, and mice^23^, to investigate the pathogenesis of glaucoma. Primary TM cells are a powerful tool for understanding glaucoma. However, in vitro models cannot simulate the complex dynamics within in vivo conditions, including constant exposure to the flow of aqueous humor and the anatomical and biochemical surroundings. Therefore, animal models play an essential and irreplaceable role in glaucoma research. In recent years, murine models have become very popular in glaucoma research, with mouse genetics facilitating the ability to modify gene expression in a specific tissue. The TM of the mouse resembles the human trabecular meshwork (hTM) in morphology for the most part^24^ and in gene expression shown by single-cell RNA sequencing^25^. Another advantage of mouse glaucoma models is that pathways for triggering glaucoma are similar to human pathogenesis.

Glucocorticoids are extensively used as anti-inflammatory drugs that have been reported to induce secondary iatrogenic open-angle glaucoma. The elevated IOP is caused by changes in the TM, striking similar to changes seen in POAG^16^. Mice treated with dexamethasone via osmotic minipumps^26^ show ultrastructural changes of TM similar to their human counterpart^24^. Other applications have been established, including topical application via eye drops^27^ or periocular injections of DEX 21-acetate^28^, resulting in an elevated IOP and reduction in the aqueous outflow facility. Another option to induce glaucoma in mice is the usage of TGFB2, a profibrotic cytokine elevated in the aqueous humor of patients with POAG^29,30^. Shepard et al. increased TGFB2 release in aqueous humor by adenoviral gene transfer of a bioactivated form of TGFB2. The IOP rose significantly and induced glaucomatous optic neuropathy^31^.

One problem concerning murine glaucoma models is the lack of knowledge about the comparability between hTM and murine trabecular meshwork (mTM) cells regarding their response under pathological conditions. To our knowledge, the availability of primary mTM cells for cell culture has been limited because of difficulties in isolation. Two different techniques for TM cell isolation in mice have been described prior. TM cell isolation using magnetic beads relies on their phagocytic properties^23^ or the microdissection of the trabecular meshwork with an additional cleaning step in cell culture^32^.

We recently developed an easy-to-perform and reliable technique to isolate mTM cells^33^, enabling us to perform this present in vitro study characterizing and comparing hTM with mTM cells under simulated pathological conditions. For this purpose, the cells were exposed to TGFB2 or Dex, which leads to epithelial-to-mesenchymal transition (EMT) of TM cells. We aim to assess the robustness and transferability of glaucoma models in mice to evaluate new treatment approaches, such as targeted restoration of physiological outflow resistance of the TM in vivo.

## Materials and methods

### Materials

**Table:** 

Materials	Sources	Identifiers
Chemicals		
β-Mercaptoethanol	Sigma-Aldrich	#M6250
AlexaFluor™488 phalloidin	Invitrogen	#A-12,379
AlexaFluor™546 goat anti-mouse	Invitrogen	#A-21,123
AlexaFluor™546 Goat anti-Rabbit	Invitrogen	#A-11,035
Bovine Serum Albumin (BSA)	Carl Roth	#T844.2
Collagen IV Rabbit pAb	Abcam	#ab6586
Dexamethasone (DEX)	Sigma-Aldrich	#D4902-25 mg
Dublecco`s Phosphate-buffered Saline (PBS)	Lonza	#BE17-512 F
Eagle’s Minimum Essential Medium (MEM)	Sigma-Aldrich	#M8042-500ML
Ethanol	Carl Roth	#5054.1
Fetal Bovine Serum (FBS)	Pan-Biotech	#P40-37500
Fibronectin Monoclonal Antibody (FBN11)	Fisher Scientific	#11,324,553
Fibronectin/FN1 Rabbit mAb	Cell Signaling	#26,836
Fluorescent Yellow Particles	Sperotech	#FL-2052-2
Gelatin from cold water fish skin	Sigma-Aldrich	#G7765-250ML
GlutaMAX™	Gibco	#3505- 061
goat anti-mouse IgG-HRP	Santa Cruz	#sc-2005
Goat serum	Millipore	#S26-100 ML
Hank’s Balanced Salt Solution (HBSS)	Biowest	#L0605
Laemmli Sample Buffer	BioRad	#1,610,747
Milk powder	Carl Roth	#T145.2
Myocilin Mouse mAB	Sigma-Aldrich	MABN866
Penicillin-Streptomycin (Pen-Strep)	Gibco	#15140-122
Precision Plus Protein™ All Blue	BioRad	#161–0373
Recombinant Human TGFB2	Peprotech	#100-35B
Roti^®^-Mount FluorCare DAPI	Carl Roth	#HP20.1
ROTI^®^Fair TBS 7.6	Carl Roth	#1244.2
RotiÒ Histofix 4%	Carl Roth	#P087.4
Starbright™ Blue 700 goat anti-rabbit antibody	BioRad	#12,004,162
SuperSignal™ West Pico PLUS Chemiluminescent Substrate	Thermo Fisher	#34,579
TGX Stain-Free™FastCast™ Acrylamide Kit, 7.5%	BioRad	#1,610,181
Transblot Turbo 5X Transfer Buffer	BioRad	#L002051F
Transblot Turbo Mini Size LF PVDF Membrane	BioRad	#L002047A
Transblot Turbo Mini Size PVDF Membrane	BioRad	#L002045A
Transblot Turbo Mini size transfer stacks	BioRad	#L002043B
Triton™X-100	Sigma-Aldrich	#X100-100ML
TrypLe™ Express Enzyme	Gibco	#12604-021
Tweenâ 20	Sigma-Aldrich	#P9416-50ML
Vimentin (VIM) Rabbit mAb	Cell Signaling	#5741S
α-Smooth Muscle Actin (D4K9N) XP^®^ Rabbit mAb	Cell Signaling	#19,245
Fibronectin from bovine plasma	Sigma-Aldrich	#F1141
Human donor tissue		
Eye	Hannover Medical School	
Animals		
C57BL/6J mice	ZTL MHH	
Instruments/software		
6 well cell culture plate	Greiner bio-one	657 − 160
12 mm diameter microscope slide coverslips	Glaswarenfabrik Karl Hecht	#41,001,113
24 well plates	Sarstedt	#83.3922.300
BioTek Lionheart™ FX Automated Microscope	Agilent	www.agilent.com
ChemiDoc MP Imaging System	BioRad	www.biorad.com
Gen5 Image Prime 3.05	Agilent	www.agilent.com
GraphPad Prism 5	GraphPad	www.graphpad.com
IBM SPSS Statistics 28	IBM	www.ibm.com
Image Lab 6 software	BioRad	www.biorad.com
Microsoft Excel 2019	Microsoft Corporation	www.microsoft.com
Orbital shaker	Heidolph	Rotamax 120
Stereo Microscope	Leica	M80
Spark ^®^ Multimode Microplate Reader	Tecan	www.tecan.com
Zeiss Axio Observer Microscope	Carl Zeiss	Z1
ZEN-Blue Microscopy Software	Carl Zeiss	

### Methods

#### Primary mTM cell isolation

Primary TM cells were isolated from three five-month-old C57BL/6J mice, which were sacrificed using cervical dislocation and enucleated directly afterward. The animals were obtained from our breeding facility (ZTL MHH, Hannover, Germany). All housing and experimental procedures complied with the German Animal Welfare Act, regulations for laboratory animals, the Laboratory Animal Register Law, and Directive 2010/63/EU of the European Parliament and the Council regarding the protection of animals used for scientific purposes. Additionally, we adhered to the ARVO Statement for the Use of Animals in Ophthalmic and Vision Research. The TM was isolated as previously described^33^. Following a cervical dislocation, eyes were enucleated, underwent a brief immersion in 70% ethanol for 30 s, and were then transferred to a Petri dish filled with Hank’s Balanced Salt Solution (HBSS). All following manipulations were conducted under a dissecting microscope.

A scalpel and scissors were employed to bisect the eyes anterior-to-posteriorly. Then, the vitreous and lens were carefully excised. Next, the posterior portion of the eyeball was discarded by making an incision near the equator. The iris and attached ciliary body were gently detached by pulling it away using forceps. The outflow tissue was identified as a pigmented circumferential strip against the transparent cornea. Subsequently, the pigmented TM strip and sections of the cornea were isolated without causing harm to the outflow tissue utilizing a scalpel. The dissected cornea/TM strips were placed in the center of one well of a 6-well tissue culture plate, ensuring the pigmented side faced downward. Excess HBSS surrounding the cornea/TM strips were discarded, followed by air-drying for 3 to 5 min at room temperature.

TM cell nutrient medium was added dropwise into the 6-well until the cornea/TM strips were submerged but securely adhered. MEM was supplemented with 1% GlutaMAX™, 10% FBS, and Pen-Strep as TM cell growth medium. Cornea/TM strips were cultured using a CO2 incubator at 37 °C for 3–4 days to facilitate proper attachment. Once the cells occupied approximately 20–30% of the surface area, typically within the first week of cell proliferation, the cornea/TM strips were cautiously removed with forceps to prevent the outgrowth of non-pigmented cell types. After TM cells reached around 90% confluency, cells were passaged and used in the experiments. Cells ranging from passages 2 to 5 were used. After passage 6, cells were discarded because of changes in morphology and loss of typical characteristics.

#### Primary hTM cell isolation

Eyes were enucleated for therapeutic reasons in the operating room of our clinic and donated by two different patients suffering from Phthisis bulbi. The procedures were carried out following the tenets of the Declaration of Helsinki, following the guidelines and regulations of and with the approval of the ethics committee of Hannover Medical School (Study No. 99931_BO_K_2021). Written informed consent was obtained from both subjects. Shortly after enucleation, the eyeballs were sterilized by immersion in 70% ethanol for 30 s. The isolation of the hTM was performed under a dissecting microscope using forceps as previously described^34^. Therefore, whole globes were divided near the equator using a scalpel. The iris, lens, and ciliary processes were delicately excised using a scalpel as one unit from the anterior segment. The Schlemm’s Canal and filtering portion of the TM could be visualized utilizing the dissection microscope.

The hTM tissue was isolated via blunt dissection. One tip of fine-tipped forceps was inserted into the lumen of the Schlemm’s Canal and the other on top of the anterior TM. Applying gentle tension to the TM tissue separated it from the scleral spur, Schwalbe’s line, and Schlemm’s Canal. The isolated cornea/TM strip was then transferred to the center of one well of a 6-well plate. Sufficient medium was carefully added to cover the TM strip with medium without allowing it to float away. An outgrowth of TM cells from the isolated strip of the Schlemm’s canal could be observed after one week of initial culture. After a substantial population of Tm cells outgrew, they were passaged and used for the experiments. HTM cells ranging from passages 2 to 6 were used. After passage 7, cells changed their morphology and characteristics and were discarded.

#### Cell culture

Primary hTM and mTM cells were grown in MEM supplemented with 1× GlutaMAX™, 10% FBS, and 1× Pen-Strep. For TGFB2 and DEX treatments, the same media with 2% FBS concentration was used. Passaging of primary cells was performed by using TrypLe Express.

#### Bright-field microscopy

Bright-field and phase-contrast microscopy of TM cells in culture were conducted 1 and 2 weeks after placing the isolated cornea/TM strip in one well of a 6-well plate. The Microscope images were taken using an Observer Z.1 microscope using ZEN-Blue analysis or the BioTek Lionheart™ FX automated microscope.

#### Detection of phagocytosing cells with fluorescent microbeads

A total of 5 × 10^4^ TM cells were cultured on microscope slide cover slips placed in individual wells of a 24-well plate. 2 µl of Fluorescent Yellow Particles was administered to each well after an initial incubation period of 24 h. Following a 48-hour incubation, the medium was removed, and cells were subjected to four Dublecco`s Phosphate-buffered Saline (PBS)) washes before fixation for immunostaining. The methodology followed the procedures described below for immunocytochemistry (ICC) staining but was modified by incorporating only 1:2000 AlexaFluor™ Plus 555 phalloidin.

The percentage of cells engaging in phagocytosis of fluorescent microbeads was evaluated using the BioTek Lionheart™ FX automated microscope and analyzed with Gen5 Image Prime 3.05 software. A 4 × 4 composite image, incorporating DAPI, green (GFP, for detecting fluorescent microbeads), and red (RFP, for detecting Filamentous-Actin (F-actin)) channels, was captured of each coverslip using a 10× objective. These 16 images were merged into one unified image for subsequent analysis. In the DAPI channel, DAPI-stained nuclei were utilized as primary masks for overall cell quantification, employing an object threshold exceeding 5000 and a size ranging from 8 to 45 μm. Secondary masks were created by expanding the primary masks in the GFP channel with a 20 μm margin. Objects with a peak GFP signal exceeding 2920 were identified as GFP-positive and counted. The percentage of cells engaging in phagocytosis of fluorescent microbeads was determined by dividing the number of GFP-positive cells by the count of DAPI-stained nuclei. These outcomes were derived from three biological replicates, each with two technical replicates.

#### TGFB2 and DEX treatment

For ICC, TM-cells were seeded in 24-well plates, each containing 5 × 10^4^ in 500 µl of the medium. For Western blot analysis, 1.5 × 10^5^ cells in 1.5 ml of medium were seeded in 6-well plates. Cell density was chosen to reach 80–90% confluence within the cell culture after ten days of treatment. The medium was changed every three days. In the TGFB2-treated group, cells were exposed to 20 ng/ml recombinant human TGFB2. In the DEX exposed group, DEX stock solution consisting of 1 mg DEX dissolved in 1 ml of pure ethanol and 49 ml of MEM 2% FBS was added, reaching a concentration of 500 nM DEX in the medium. DEX concentration was chosen because preliminary tests showed no additional reaction above 500 nM in ICC. The resulting ethanol concentration of 0.03% in the cell culture was negligible and not accounted for in the control or TGFB2 treatment. Cells were treated for 7–14 days, with an average of 10 days of treatment. At least three biological replicates differing in passage number and origin were performed.

#### ICC staining

5 × 10^4^ cells were seeded on 12 mm coverslips in 500 µl MEM containing 2% FBS in each well of a 24-well plate. After treatment, cells were washed twice with 1× PBS and immediately fixed with 4% Roti^®^Histofix for 1 h at room temperature. Cells were washed twice with 1× PBS before blocking for 1 h at room temperature with a blocking solution. The blocking solution contained 2% goat serum, 1% Bovine Serum Albumin (BSA), 0.1% cold fish skin gelatin, 0.1% Triton™X-100, and 0.05% Tween^®^ 20 dissolved in 1× PBS. HTM cells were incubated with SMA Rabbit mAb, Vimentin (VIM) Rabbit mAb, Fibronectin (FN) 1 Rabbit mAb, Collagen (COL) IV Rabbit pAb, and Myocilin (MYOC) Mouse mAb diluted 1:1000 in blocking solution at 4°C overnight. For mTM, the same antibodies were used except for FN1. FN1 Rabbit mAb showed non-specific binding patterns within mTM cells; therefore, Fibronectin Monoclonal Antibody (FBN11) was used instead. Afterward, cells were washed twice with PBST, a solution out of 0.1% Tween^®^ 20 in 1× PBS for 10 min. Cells were further incubated with 1:1000 AlexaFluor™546 Goat anti-Rabbit and 1:1000 AlexaFluor™488 phalloidin. Cells were incubated with murine primary antibodies and incubated with 1:1000 AlexaFluor™488 phalloidin and 1:1000 AlexaFluor™546 goat anti-mouse for two hours at room temperature.

After 3 × 10 min washing steps in PBST, the coverslips were mounted upside down on a microscope slide with Roti^®^-Mount FluorCare DAPI. Microscope images were taken using an Observer Z.1 microscope using ZEN-Blue analysis software.

#### Fibronectin coating for ICC

12 mm diameter microscope coverslips were placed in each well of a 24-well plate. Subsequently, 250 µl of fibronectin solution containing 5 µg of fibronectin from bovine plasma dissolved in sterile HBSS was added to each well to coat the coverslips. Plates were incubated for 2 h at 37 °C. After discarding the fibronectin solution, plates were washed two times with HBSS to remove access fibronectin. 5 × 10^4^ cells were then seeded in 500 µl MEM containing 2% FBS in each well of a 24-well plate. All the following steps were performed as described above.

#### Western blot

TM-cells were lysed with 1× Laemmli Sample Buffer containing 10% of β-Mercaptoethanol. Samples were then denatured for 5 min at 95 °C and stored at -20 °C. 7.5% SDS-Gels were cast using TGX Stain-Free™FastCast™ Acrylamide Kit 7.5% for SDS-Page. Afterward, all samples were normalized to total protein. Therefore, 10 µl of each sample was applied to each lane and run at 120 Volts for approximately 60 min. Next, the blot was performed as described below, and the images were imported into Image Lab 6 software for total protein normalization. Adapted sample volumes were then used to perform a second SDS page. Transblot Turbo Mini size transfer stacks and membranes were soaked in Transblot Turbo 5X Transfer Buffer diluted according to manufacturer protocol. For the FN1 Western blot, Transblot Turbo Mini Size PVDF Membrane was used to perform the blot at 1.3 A, 25 volts, for 10 min. For the Actin-alpha-2 (ACTA2) Western blot, Transblot Turbo Mini Size LF PVDF Membrane was used, performing the blot at 1.3 A, 25 Volts for 7 min. Next, the PVDF membrane was washed three times in TBST (TBS + 0.1% Tween 20) for 5 min and blocked with 5% milk powder in TBST at room temperature (RT) for one hour. The primary antibodies FN1 Mouse mAb or SMA Rabbit mAb were diluted 1:1000 in 5% BSA in PBST. The membrane was incubated overnight at 4 °C on an orbital shaker. The following day, membranes were washed 3 × 5 min with PBST. The blots were then incubated with the secondary antibody for two hours at RT. For FN1 western blot, goat anti-mouse IgG-HRP 1:2500 was used. For the ACTA2 western blot, Starbright™ Blue 700 goat anti-rabbit antibody diluted 1:2000 in BSA 5% in PBST was used. After incubation, HRP-conjugated antibodies were detected using SuperSignal™ West Pico PLUS Chemiluminescent Substrate and the ChemiDoc MP Imaging System. Immunofluorescence was visualized directly using the ChemiDoc MP Imaging System. ImageLab 6 software was used for Western blot (WB) quantification.

#### Quantification of CLANs

CLAN quantification treatment with DEX/TGFB2 and ICC for F-Act was performed as described above. Nuclei were stained using Roti^®^Mount FluorCARE DAPI. Microscopic images were taken using 20× magnification and used to evaluate CLAN formation. CLAN-positive cells had to contain at least one CLAN with a minimum of three triangles^23^. The ratio of CLAN-positive cells to total DAPI-stained cells for each group was calculated for at least 10 different biological replicates, investigating 5 randomly assigned regions per replicate.

#### Statistical analysis

GraphPad Prism 9 and Microsoft Excel 2019 were used for the statistical analysis. Each data is expressed as mean ± Standard Deviation (SD). Analysis was performed with unpaired Student *t*-tests. A p-value of < 0.05 was considered statistically significant.

## Results

### Basic morphology and observations during cell culture

**Fig. 1:**
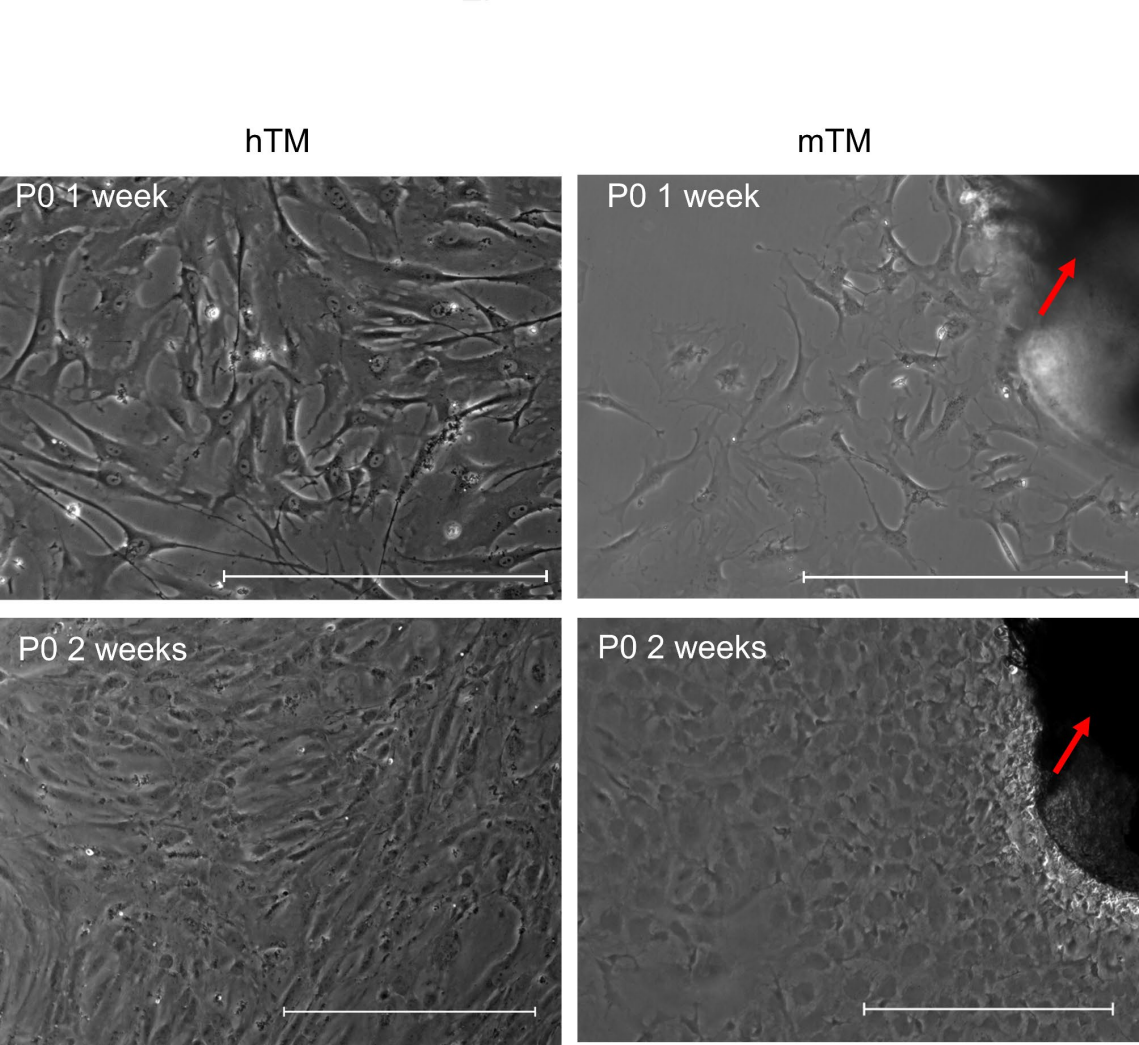
Representative phase-contrast images of hTM and mTM one and two weeks after initial culture. Left column: Phase-contrast image for hTM at P0 after 1 and 2 weeks in cell culture. Right column: Phase-contrast for mTM at P0 after 1 and 2 weeks in cell culture. Red arrows mark the residual cornea/TM stripe. The scale bar represents 500 μm.

The TM stripe of both species was isolated by microsurgery and cultured in one well of a 6-well plate. Media was exchanged every 3 to 4 days. An outgrowth of cells from the TM isolate could be observed mainly within the first week, ranging up to three weeks. Once confluent, cells showed a cobblestone-like pattern with numerous cell extensions resembling endothelial-like phenotypes. HTM and mTM cells (Fig. 1) showed a similar morphology in bright field microscopy during outgrowth and after 1 week in cell culture. After 2 weeks in cell culture, the similarities were less pronounced, with hTM cells developing increasingly directional growth patterns and cells elongating. Cells were passaged when reaching 80–90% confluence and discarded after passage 5 for mTM cells and passage 6 for hTM cells. Cells with higher passage numbers changed towards a mesenchymal phenotype, shown by increased expression of F-Act orientated in parallel arrays and elongated cell bodies for both mTM and hTM cells, as described by others^34^.

### Phagocytic properties of hTM and mTM cells

The distinctive phagocytic properties observed in hTM cells^35^ and mTM cells^36^ are a reliable marker for distinguishing TM cells from neighboring cells. Decreased phagocytic activity due to steroid treatment has been shown for hTM cells^37^. Decreased phagocytosis leads to increased extracellular matrix deposition within the trabecular meshwork’s outflow pathway and, therefore, has been linked to elevated intraocular pressure^10^. The similarities between the phagocytic properties of human and murine TM cells have not been quantified yet.

Subsequently, an assessment of hTM and mTM cell phagocytic properties was carried out. Cell culture was performed on coverslips, followed by exposure to fluorescent microbeads for 48 h. Before fixation, the microbeads were meticulously washed to remove non-phagocytosed beads. Bead phagocytosis by TM cells was confirmed by fluorescence staining with Alexa555-Phalloidin and the nuclear stain DAPI, as described above.

**Fig. 2:**
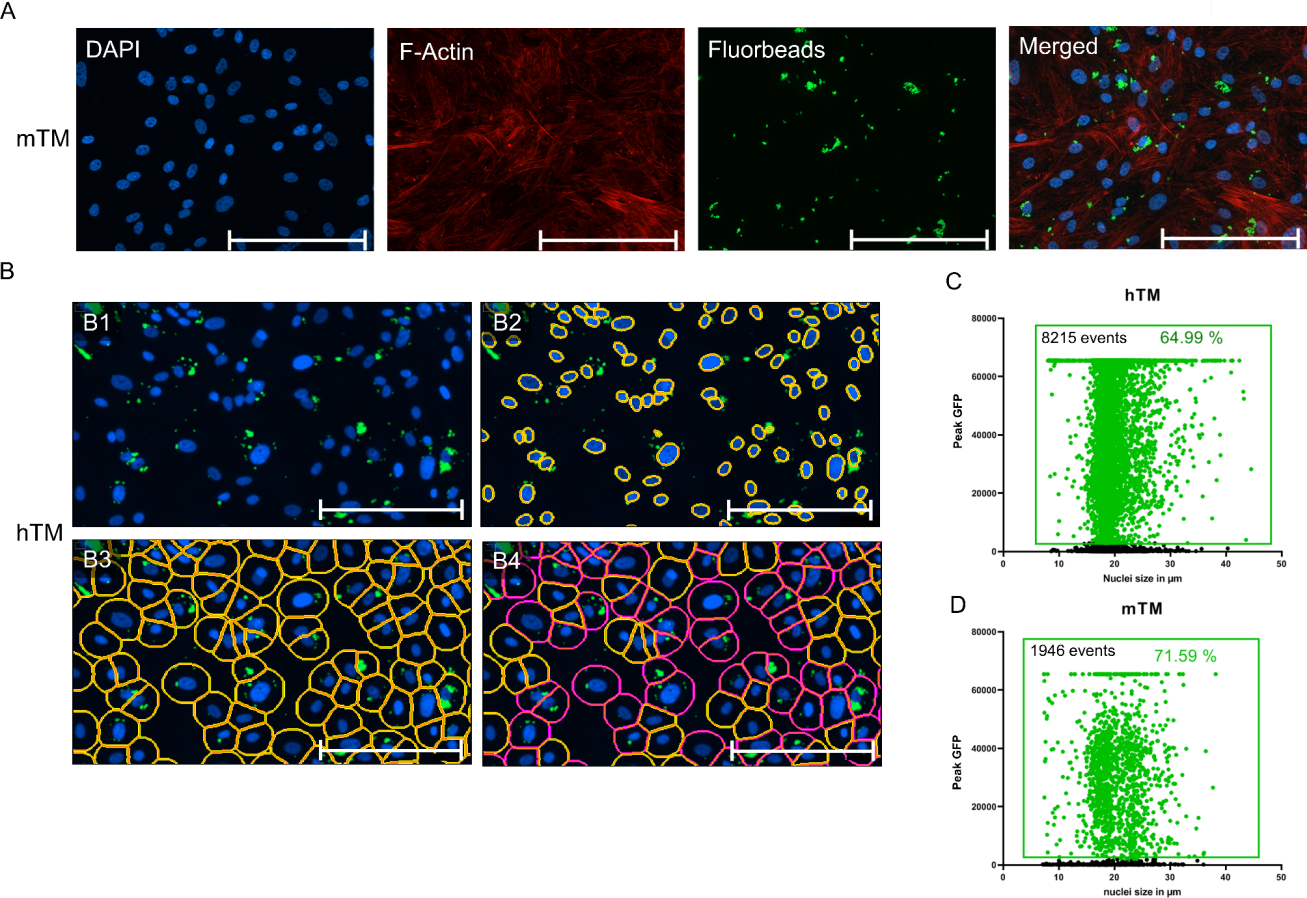
Identification and quantification of phagocytosing TM cells with fluorescent microbeads. (**A**) Representative ICC images of mTM cells previously exposed to green fluorescent microbeads for 48 h. Nuclei were stained with DAPI and F-actin with Alexa555-Phallodin. The scale bar represents 200 μm. (**B**) Quantitative analysis procedure of phagocytosing hTM cells using a microscope with automated XYZ stage. (**B1**) Merged image shows the cell nuclei (blue) and the fluorobeads (green) for the following analysis. (**B2**) DAPI-stained nuclei were gated in the blue channel. (**B3**) This primary mask was extended by 20 μm and used as a sub-mask in the green channel to identify fluorobeads-positive cells. (**B4**) After setting a threshold, fluorobeads-positive cells are highlighted in red, and yellow circles highlight fluorobeads-negative cells. The scale bar represents 200 μm. (**C**) XY-scatter plot for hTM cells consisting of 3 biological replicates showing the peak GFP intensity and nuclei size of 8215 analyzed cells. The fluorescent microbeads-positive counts are highlighted in green. (**D**) XY-scatter plot for mTM cells consisting of 3 biological replicates showing the peak GFP intensity and nuclei size of 1946 analyzed cells. The fluorescent microbeads-positive counts are highlighted in green.

**Fig. 3:**
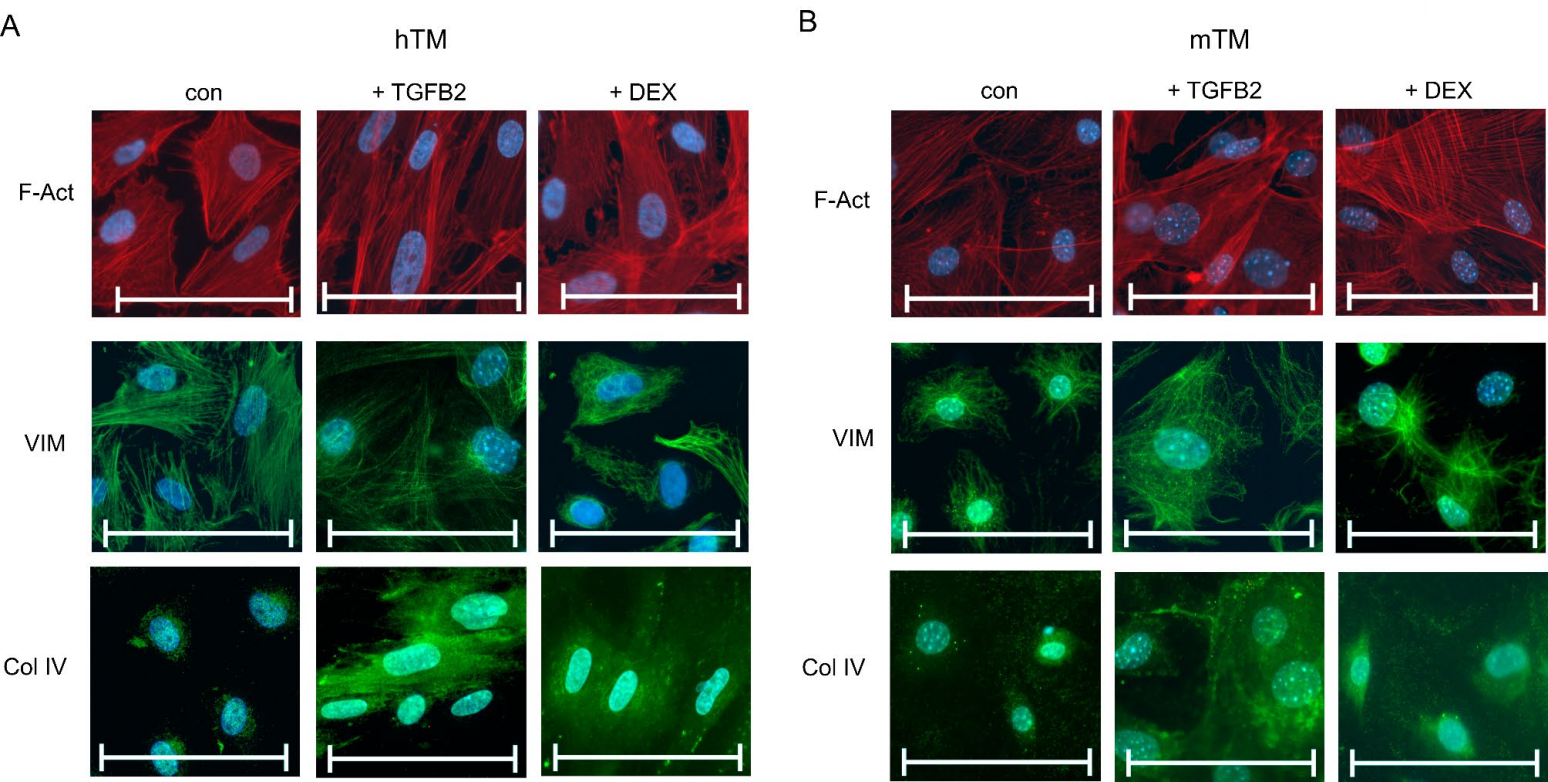
ICC staining with EMT-associated markers F-actin, vimentin, and collagen IV of hTM and mTM cells exposed for ten days without or with TGFB2 or DEX. HTM cells (**A**) and mTM cells (**B**) were immunostained for F-Act, VIM, and COL IV. The cell nuclei were stained with DAPI. The scale bar corresponds to 100 μm.

TM cells from both species displayed varying amounts of green fluorescent beads in the cytoplasm surrounding the nucleus. In order to include as many cells per well as possible in the analysis, a fluorescent microscope with an automated XYZ stage was used, and a 4 × 4 mount per coverslip with a 10× objective was taken for subsequent quantification. A composite image was created by stitching 16 individual images, and cell nuclei in the DAPI channel were gated with a primary mask (Fig. 2B3). This primary mask was expanded by 20 μm and used as a sub-mask in the green channel to identify cells containing fluorescent beads (Fig. 2B4). The analysis comprised at least three biological replicates and two technical replicates for each species. Overall, 8,215 hTM cells and 1,946 mTM cells were analyzed, of which 64.99% of hTM cells (Fig. 2C) and 71.59% of mTM (Fig. 3D) cells exhibited phagocytotic properties within the 48-hour timeframe.

### TGFB2/DEX-induced expression of F-Act, Vim, and Col4

TGFB2 has previously been reported to induce EMT-like changes in hTM cells^38^ and TM cells of cynomolgus monkeys^39^. Similar effects have been reported for hTM cells exposed to DEX^40^. Cells of epithelial phenotype undergoing EMT change to a mesenchymal phenotype. Those changes include (i) reorganizing the radially extending F-Act cytoskeleton to a parallel-orientated assembly, (ii) increased expression of ACTA2, and (iii) directional alignment of the cells. To test this, we compared unexposed hTM (Fig. 3A) and mTM (Fig. 3B) with TGFB2 or DEX-exposed counterparts for ten days. We visualized the changes in cytoskeletal structure using ICC for F-Act. In the untreated control group, hTM and mTM cells predominantly exhibited a round-like morphology. Subpopulations of more elongated cells could be shown, especially for hTM.

In contrast, hTM and mTM cells treated with 20 ng/ml TGFB2 showed elongated cell bodies and a directional intercellular arrangement of F-Act filaments. A similar effect could be observed under exposure to DEX, which also rearranged the distribution of F-Act. For hTM and mTM cells, the changes were similar.

Furthermore, we performed ICC-staining for VIM (Fig. 3), an intermediate filament protein and part of the cytoskeleton that regulates changes in cells undergoing EMT^41^. In contrast to hTM cells, untreated mTM cells showed a compact VIM network surrounding the nucleus. VIM in mTM cells spread widely throughout the cytoplasm under exposure to TGFB2 but changed only slightly under exposure to DEX. In hTM cells, VIM was spread more widely throughout the cytoplasm, and no significant changes under exposure to Dex or TGFB2 could be observed. VIM differs in its distribution for hTM and mTM cells and upon exposure to TGFB2 and DEX.

Next, we compared the ECM protein COL IV expression. COL IV has been present in the hTM cell’s basal lamina but lacks in neighboring cell types^42^. It has been reported that the hTM of patients with POAG shows increased staining for COL IV in ICC^43^. Our findings indicate that COL IV is present in untreated TM cells of both species and can be induced by DEX and even more prominent by TGFB2 (Fig. 3). COL IV accumulated close to the nuclei in the untreated control group while being widespread under exposure to TGFB2 and DEX. The structure and distribution were similar between hTM and mTM cells. Taken together, hTM and mTM cells react similarly in the control and under treatment conditions regarding basic morphology and ICC staining. TGFB2 and Dex induce an EMT in both hTM and mTM cells. The findings align with the induction of an EMT by both TGFB2 and Dex in both hTM and mTM cells.

### TGFB2/DEX-induced CLAN formation

**Fig. 4:**
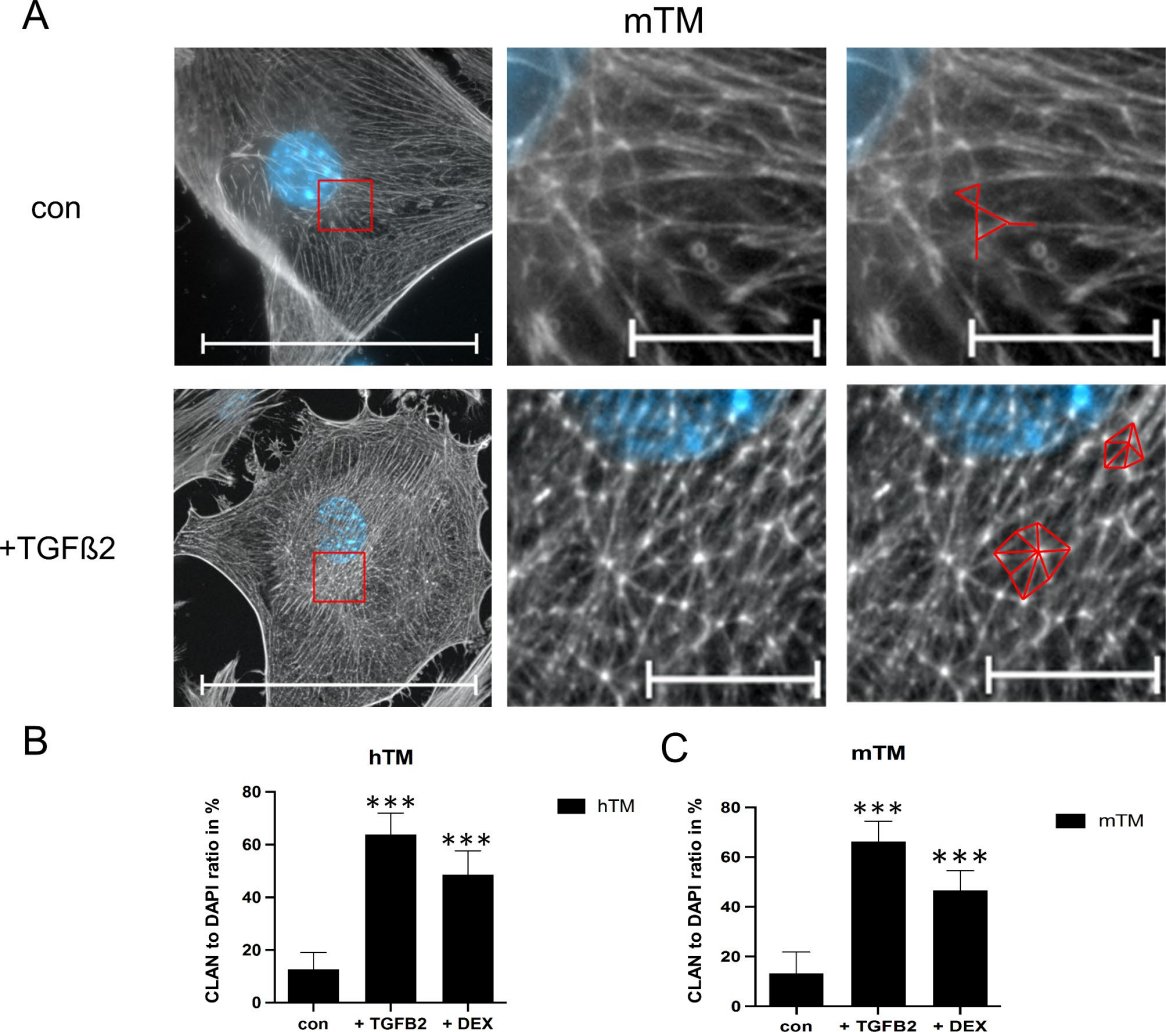
Expression of CLAN formations for confluent hTM and mTM cells after 10 days of exposure to TGFB2 or DEX. (**A**) F-Act image of mTM cell under control and TGFB2 treatment conditions. Phalloidin-A488 was previously used to stain F-Actin Filaments. The red square marks the region of interest displayed in the middle image. In the right image, the orientation of F-Actin filaments is traced by red lines. A representative CLAN formation of 7 triangles is traced for the TGFB2 treatment, while the control only shows two triangles. (**B**) The ratio of CLAN-positive to DAPI-positive hTM cells in control and under exposure to TGFB2 or DEX. (**C**) The percentage of CLAN-positive to DAPI-positive mTM cells in the control and under exposure to TGFB2 or DEX. The nuclei were stained using DAPI. The F-act channel is displayed as a monochrome image. The scale bar in the left column represents 100 μm, and the scale bar in images of the region of interest represents 10 μm. For CLAN to DAPI ratios, an unpaired two-tailed *t*-test to the control revealed significant differences (***p *<* 0.0001). The error bars display SD.

As distinct changes of F-Act under treatment conditions could be observed in ICC, we further investigated the formation of CLANs. CLANs are polygonal rearrangements in the actin cytoskeleton expressed by TM cells under stress^11^. The expression and arrangement of F-Act into CLANs increased drastically under treatment conditions, as shown for mTM cells undergoing TGFB2 treatment (Fig. 4A). The changes for hTM cells resembled those found in mTM cells. Furthermore, we analyzed the ratio of cells forming CLANs to DAPI-positive cells after exposure to TGFB2 or DEX for 10 days compared to the control (Fig. 4B, C). CLAN-positive cells had to contain at least one CLAN with a minimum of three triangles^23^. In the control group, 12.7% (*n* = 10; SD: 6.3%) of DAPI-positive hTM cells and 13.3% (*n* = 10; SD: 8.6%) of DAPI-positive mTM cells showed an expression of CLANs. After exposure to TGFB2, CLAN-positive increased to 63.9% (*n* = 10; SD: 8%) in hTM and 66.4% (*n* = 10; SD: 8.1%) in mTM cells. Under the exposure to DEX, CLAN-positive also increased but less extensively to 48.7% (*n* = 10; SD: 9%) of hTM and 46.6% (*n* = 10; SD: 7.9%) of mTM cells forming CLANs. A *t*-test confirmed the significance of the increase compared to the control (*p* < 0.001). In conclusion, hTM and mTM express CLAN formations similarly to each other in the control and under exposure to TGFB2 or DEX.

### TGFB2/DEX-induced expression of ACTA2

**Fig. 5:**
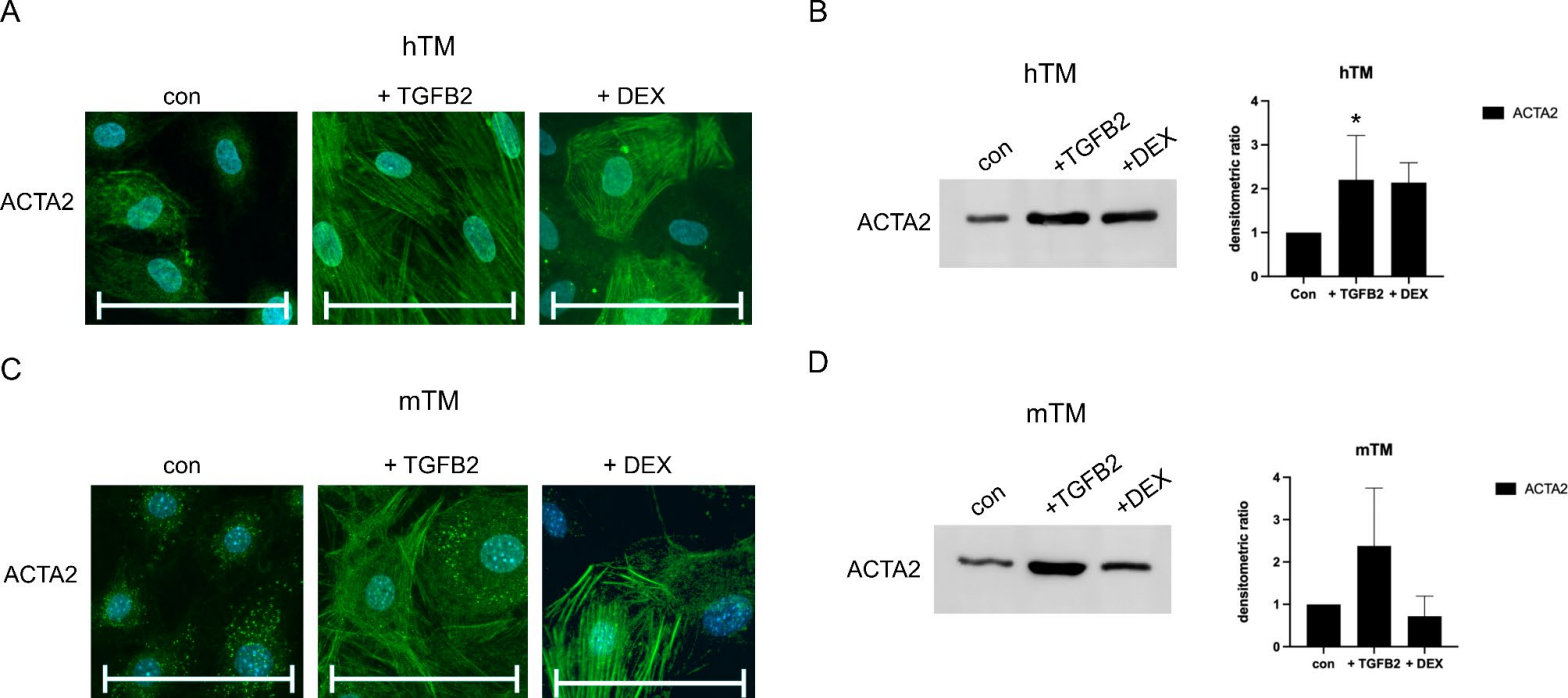
ACTA2 expression for confluent hTM and mTM cells after 10 days of exposure to TGFB2 or DEX. (**A**) ACTA2 ICC staining for confluent hTM cells in control and after ten days of exposure to TGFB2 (20 ng/ml) or DEX (500 nm). The cell nuclei were stained with DAPI. The scale bar corresponds to 100 μm. (**B**) Representative WB of hTM cells for ACTA2 and the densitometric ratio of WB for ACTA2. (**C**) ICC staining of ACTA2 on confluent mTM cells in control and after ten days of exposure to TGFB2 (20ng/ml) or DEX (500nm). The cell nuclei were stained with DAPI. The scale bar corresponds to 100 μm. (**D**) Representative WB of mTM cells for ACTA2 and the densitometric ratio of WB for ACTA2.The densitometric ratio of WB for ACTA2 was calculated from three biological replicates and normalized to total protein with further normalization to the untreated control. Corresponding total protein blots are shown in Supplemental Fig. SF1. An unpaired two-tailed **t**-test was performed on the control, revealing significant differences (*p < 0.05). The error bars display SD.

Additionally, ICC detecting ACTA2 was performed because its increased expression is typical for many cell types undergoing EMT: it increases contractility and contributes to the mesenchymal properties. In the control group ACTA2 is weakly distributed in hTM’s cytoplasm whereas in mTM ACTA2 is rather punctually distributed. Upon exposure to TGFB2 or DEX, the number of ACTA2-positive cells increased in both species, with TGFB2 causing a higher increase than DEX (Fig. 5A,C). A difference could be observed between mTM and hTM cells, with mTM cells responding more uniformly to the exposure, resulting in increased staining within all cells (Fig. 5C). HTM cells could be divided into responders and non-responders, with some cells showing intense ACTA2 staining and others showing no response within the same cell culture (Fig. 5A).

Next, WB analysis was then performed for ACTA2 because only a subpopulation of hTM cells showed increased staining for ACTA2 under exposure to TGFB2 or DEX in ICC. ACTA2 was detected as a single band at 42 kDa (Fig. 5B,D). The evaluation of the WB revealed an increase of ACTA2 by both hTM (Fig. 5B) and mTM (Fig. 5D) cells under exposure to TGFB2. The densitometric ratio was normalized to total protein and further normalized to the non-treated control (Supplemental Fig. A). The analysis resulted in a significant densitometric ratio increase of 2.20 (*N* = 3, SD = 0.82) for hTM and a non-significant increase of 2.38 (*N* = 3, SD = 1.37) for mTM cells under exposure to TGFB2 (Supplemental Fig. D).

Exposure to Dex also increased the ACTA2 protein level in hTM cells (Fig. 5B), whereas mTM cells produced a consistent amount of ACTA2 (Fig. 5D). The analysis performed as described above resulted in a densitometric ratio of 2.14 (*N* = 3, SD = 0.45) for hTM and 0.72 (*N* = 3, SD = 0.48) for mTM cells under the exposure to TGFB2 (Supplemental Fig. D).

### TGFB2/DEX-induced expression of FN1

**Fig. 6:**
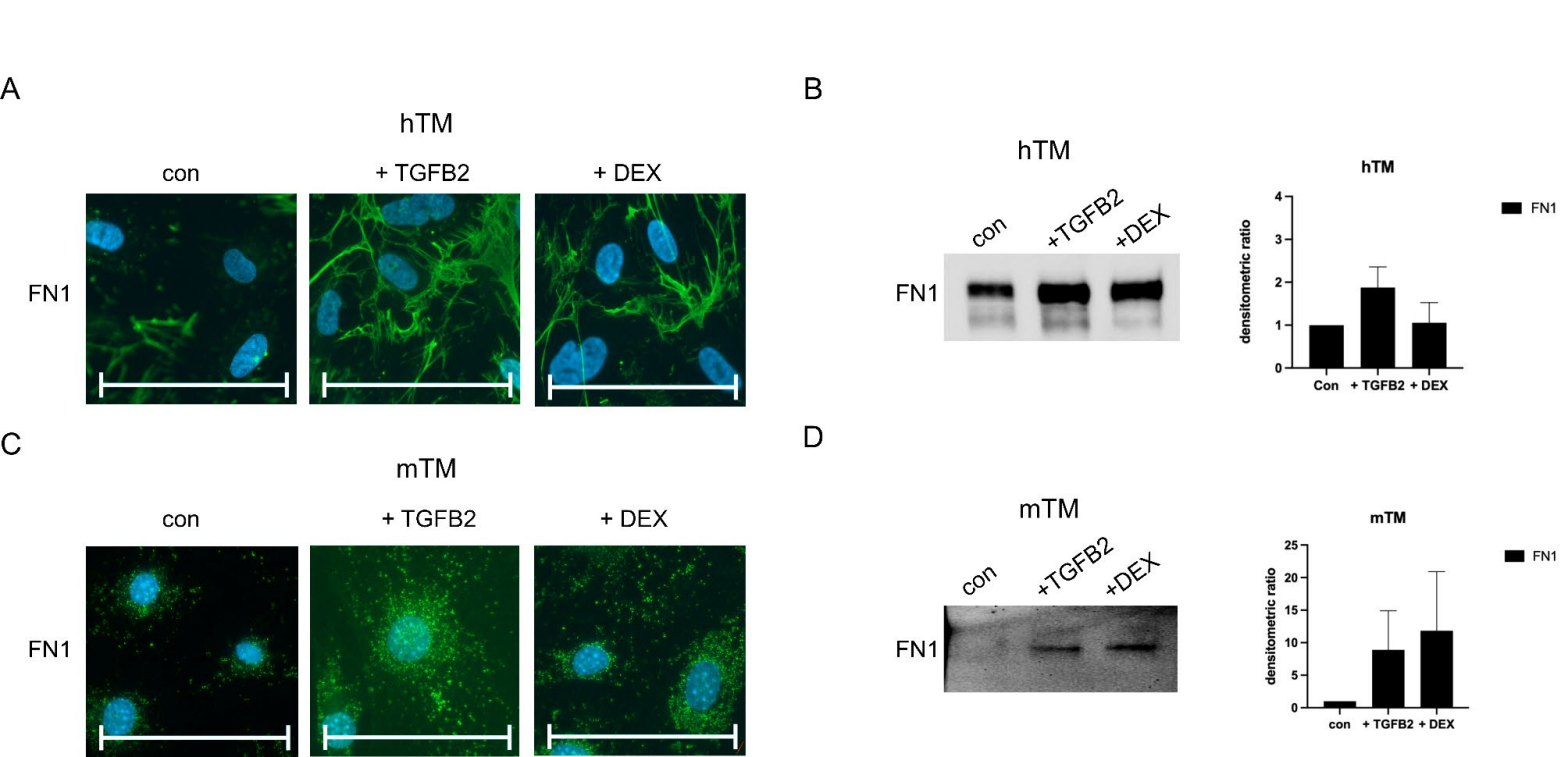
FN1 expression for confluent hTM and mTM cells after 10 days of exposure to TGFB2 or DEX. (**A**) FN1 ICC staining for confluent hTM cells in control and after ten days of exposure to TGFB2 (20 ng/ml) or DEX (500 nm). The cell nuclei were stained with DAPI. The scale bar corresponds to 100 μm. (**B**) Representative WB of hTM cells for FN1 and the densitometric ratio of WB for ACTA2. (**C**) FN1 ICC staining for confluent mTM cells in control and after ten days of exposure to TGFB2 (20 ng/ml) or DEX (500 nm). The cell nuclei were stained with DAPI. The scale bar corresponds to 100 μm. (**D**) Representative WB of mTM cells for FN1 and the densitometric ratio of WB for FN1. The densitometric ratio of WB for FN1 was calculated from three biological replicates and normalized to total protein with further normalization to the untreated control. The corresponding total protein blots are shown in Supplemental Fig. SF1. An unpaired two-tailed *t*-test to the control was performed (*p < 0.05). The error bars display SD.

FN1 is a prominent part of hTM ECM and might play a role in regulating outflow resistance^42^. TGFB2 induces FN1 expression in hTM cells as part of EMT^44^. Therefore, we performed ICC for FN1. FN1 was expressed by untreated hTM (Fig. 6A) and mTM cells (Fig. 6C), with TGFB2 and DEX inducing its expression. TGFB2 caused higher levels of FN1 in both species. The structure of FN1 differed between hTM and mTM cells, with FN1 in hTM cells consisting of long strands overlaying cell borders and complex formation, while FN1 in mTM cells stained as accumulations of dots close to the nuclei.

The significant differences in ICC staining were subsequently quantified using WB analysis. FN1 protein was detected as a single band at 220 kDa (Fig. 6B,D). Longer exposure times were necessary to detect bands of murine samples (Fig. 6D), indicating expression at lower levels than hTM samples.

The evaluation of the WB analysis showed an increase of FN1 by both hTM (Fig. 6B) and mTM (Fig. 6D) cells under exposure to TGFB2 and Dex, with TGFB2 having a more substantial effect on hTM cells. The densitometric ratio was normalized to total protein and further normalized to the non-treated control (Supplemental Fig. B,C). The analysis revealed a densitometric ratio of 1.88 (*N* = 3, SD = 0.48) for hTM and 8.89 (*N* = 3, SD = 6.01) for mTM cells under exposure to TGFB2 (Supplemental Fig. D).

Exposure to Dex also increased the FN1 protein level in both species (Fig. 6B,D). The analysis performed as described above resulted in a densitometric ratio of 1.06 (*N* = 3, SD = 0.47) for hTM and 11.83 (*N* = 3, SD = 9.08) for mTM cells under the exposure to Dex (Supplemental Fig. D).

### TGFB2/DEX-induced expression of MYOC

**Fig. 7:**
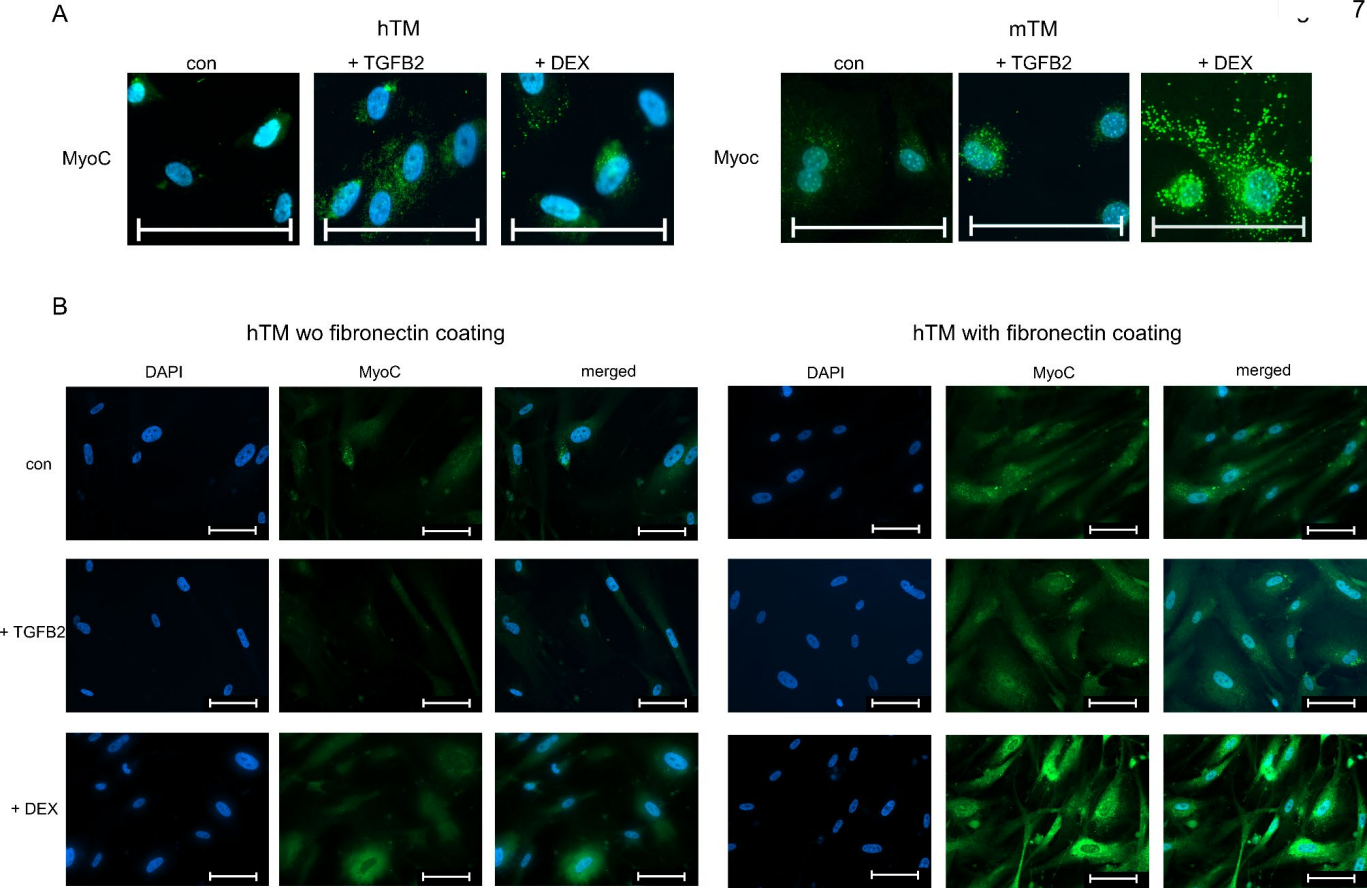
ICC staining of MYOC for confluent hTM and mTM cells after 10 days of exposure to TGFB2 or DEX. (**A**) HTM and mTM cells were immunostained for MYOC. The DAPI and MYOC channels are merged. (**B**) HTM cells immunostained for MYOC without (left side) and with (right side) fibronectin coating. DAPI and MYOC channels are displayed individually and merged. The cell nuclei were stained with DAPI. The scale bar corresponds to 100 μm.

Finally, ICC for MYOC was conducted, which has been reported to increase under exposure to DEX^45^. MYOC is a standard identification criterion for TM cells in both species and differentiates them from neighboring cells ^23,46^. In our experiments, in both mTM and hTM cells, the expression of MYOC was induced by DEX and TGFB2, whereas DEX had a more substantial effect (Fig. 7A). The control staining for MYOC was barely visible in both species. The intensity of staining for MYOC differed between hTM and mTM cells. In hTM cells, MYOC staining was less intense and was only close to the nucleus. In mTM cells, MYOC staining was more prominent and widespread throughout the cytoplasm. Because results between hTM and mTM cells differed, we optimized the protocol for hTM cells by coating the coverslips with fibronectin before usage in cell culture. This procedure resulted in an elevated expression of MYOC and a very similar structure to MYOC in hTM cells (Fig. 7B).

## Discussion

Murine glaucoma models facilitate a powerful tool in glaucoma research. The transferability of murine glaucoma models is based on similarities in morphology^24^ and gene expression shown by single-cell RNA sequencing^25^.

Our study aimed to broaden the knowledge base for assessing the transferability of murine glaucoma models by comparing the behavior of hTM and mTM cells under simulated pathological conditions in vitro. Therefore, we evaluated changes in the ECM and cytoskeleton of hTM and mTM cells under exposure to TGFB2 and DEX. Elevated concentrations of TGFB2 have been found in aqueous humor samples from patients with POAG^29^, while medical treatment with DEX is linked to the pathogenesis of secondary iatrogenic open-angle glaucoma^16^. The findings of our experimental study provide evidence that hTM and mTM cells react very similarly under certain conditions. TGFB2 and DEX-induced EMT-like changes in mTM cells have been described for hTM cells^38,40^. ICC for F-Act and VIM were performed to assess changes in the cytoskeleton assembly during EMT, and similar results were also found for both cell types. A significant increase of CLANs, as described for hTM cells under DEX exposure^17^ and bovine TM cells under TGFB2 exposure^18^, could also be observed in both cell types. ECM components of the TM seem to match mostly with Col IV and ACTA2 being expressed at similar levels and induced by DEX and TGFB2 exposure.

Additionally, the phagocytosis of fluorescent microbeads by hTM and mTM cells in cell cultures showed a remarkable resemblance.

Besides the similarities, our study indicates certain limitations in comparing hTM and mTM cells. While most components of the ECM were expressed by both hTM and mTM cells at similar levels, FN1, a primary compound of the ECM of hTM cells^42^, seems to exist only at low levels in murine TM cells. How FN1 influences outflow resistance has not been investigated yet. However, research on other cell types indicates that FN1 might influence ECM turnover, production, gene expression, and cytoskeletal organization, potentially affecting outflow resistance^47^.

MYOC could be detected by ICC and WB in both species and was induced by Dex and TGFB2, with Dex showing a more significant effect. Nevertheless, ICC for MYOC implies that hTM cells expressed MYOC at lower levels in vitro. Only after coating coverslips with fibronectin^48^, protein levels and patterns are aligned between hTM and mTM cells. This observation challenges our findings of substantially higher FN1 expression by hTM cells compared to mTM cells confirmed by Western blot analysis. In mTM cells exposed to DEX, low levels of FN1 correspond with high levels of MYOC (Fig. 6C + D and Fig. 7A). In hTM cells, high levels of FN1 correspond with low levels of MYOC (Fig. 6A + B and Fig. 7A), but supplying hTM cells with a fibronectin coating of the culture vessel increases MYOC expression (Fig. 7B).

Our findings could be explained by differing effects of endogenously produced FN1 in the TM’s ECM and additionally supplied fibronectin coating derived from bovine serum on the hTM cells.

The biological activity of fibronectin is highly modular, and its structure is flexible. It is a soluble protein with many biological domains within the aqueous humor, similar to fibronectin in plasma^49^. As part of the TMs, FN1 transforms into an insoluble fibril ECM with accessible biological domains^49^. Therefore, FN1coating could facilitate hTM cells’ MYOC production by allowing proper attachment to their surroundings^47^ without occupying a function similar to endogenously produced fibronectin fibrils. This effect needs to be further investigated and quantified.

Different cell characteristics could impose another limitation within the TM cell population in culture. In ICC for ACTA2, only some of the TGFB2 or DEX-exposed hTM cells showed an increased expression of ACTA2, while others did not. In contrast, hTM cells reacted more uniformly. This effect could be due to differences in the morphology of TM cells depending on their localization within the TM and the different techniques used to isolate hTM and mTM cells, which might have resulted in varying compositions of cells in cell culture. HTM cells from the anterior meshwork region and the inner uveal beams stain positively for neuronal-specific enolase. In contrast, cells of the posterior region do not^50^, and functional differences within the TM depend on localization^51^. Staining for neuronal-specific enolase could be used to identify the origin of TM cells in cell culture, eliminating this variable in further research.

While the differences found in our study are not neglectable, their significance is difficult to predict because our knowledge about the influence FN1 and MYOC have on outflow resistance and other functional aspects of the TM is limited. In contrast, the significance of the similarities regarding the ECM and cytoskeleton under EMT found in our study are well-researched and known to be directly linked to the pathogenesis of POAG and secondary iatrogenic open-angle glaucoma. Therefore, concluding the results of our study, we supplied evidence to support the transferability of murine glaucoma models to their human counterparts based on their similarities in vitro.

## Electronic supplementary material

Below is the link to the electronic supplementary material.

Supplementary Material 1

Supplementary Material 2

Supplementary Material 3

## Data Availability

Data is provided within the manuscript or supplementary information files.
