# Supplementary material for: In vitro comparison of human and murine trabecular meshwork cells: implications for glaucoma research
Source: Sci Rep. 2024 Sep 23;14:22002. doi: 10.1038/s41598-024-73057-9 (PMC11420201; doi:10.1038/s41598-024-73057-9)
Supplement: Supplementary file 1 — Supplementary Material 1 [file 41598_2024_73057_MOESM1_ESM.docx]

**Supplementary Video SV1. Time-lapse video of pigmented mTM phagocytosing pigment granules one week after initial culture**

Phase-contrast (left) and bright-field (right) movie of pigmented mTM cells growing out of the TM/cornea stip. The scale bar represents 100 µm.
