# Supplementary material for: In vitro comparison of human and murine trabecular meshwork cells: implications for glaucoma research
Source: Sci Rep. 2024 Sep 23;14:22002. doi: 10.1038/s41598-024-73057-9 (PMC11420201; doi:10.1038/s41598-024-73057-9)
Supplement: Supplementary file 2 — Supplementary Material 2 [file 41598_2024_73057_MOESM2_ESM.pdf]

Supplemtary File SF1

A

Westernblot ACTA2 (Stain Free Gel)

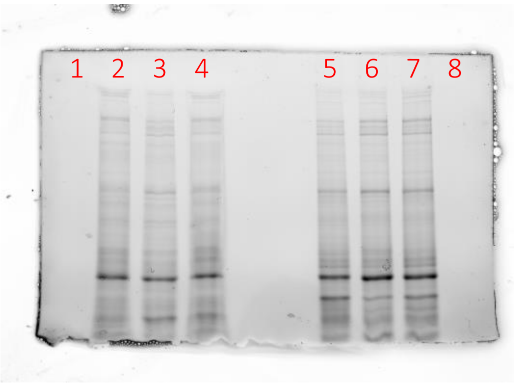

Westernblot ACTA2 (Stain Free Blot)

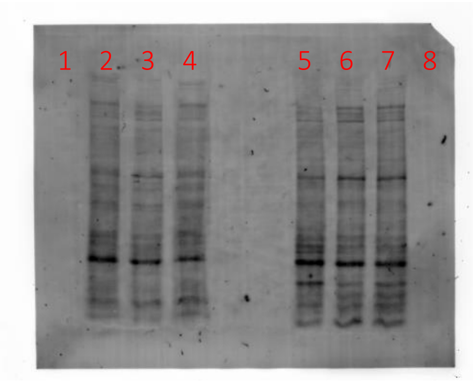

Westernblot ACTA2 (Cy5)

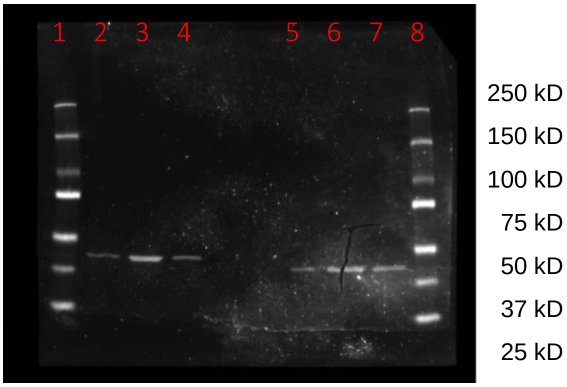

Westernblot ACTA2 (StarBright B700)

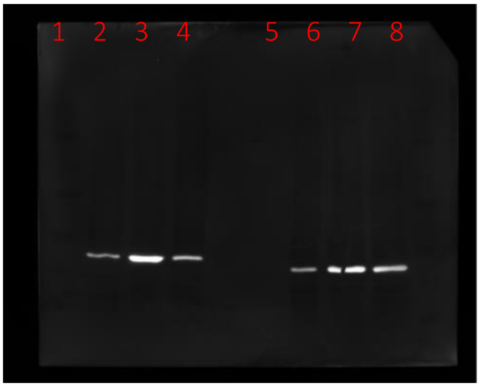

Sample:
1: Precision Plus Protein Standards All Blue
2: mTM control
3: mTM TGFß
4: mTM DEX
5:hTM control
6:hTM TGFß
7: hTM DEX
8: Precision Plus Protein Standards All Blue

B

Westernblot FN 1 hTM(Stain Free Gel)

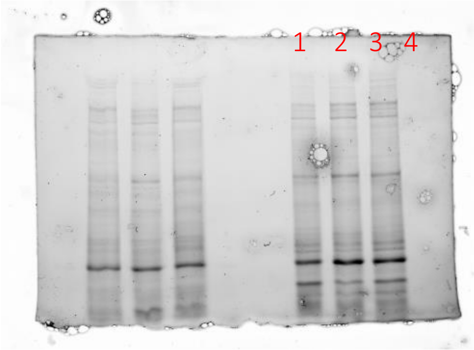

Westernblot FN1 hTM (Stain Free Blot)

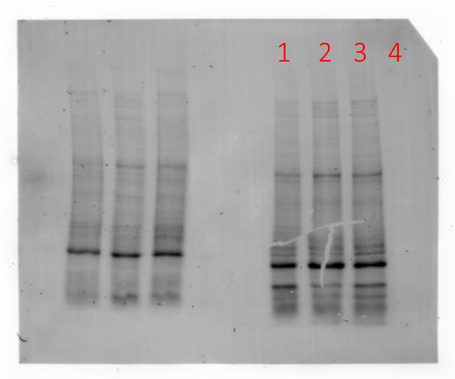

Westernblot FN1 hTM (Cy5)

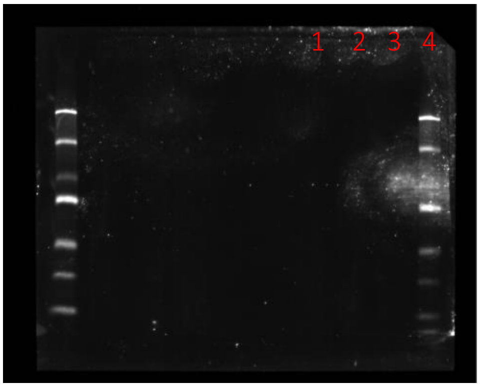

250 kD
150 kD
100 kD
75 kD
50 kD
37 kD
25 kD

Westernblot FN1 hTM (Chemiluminescence)

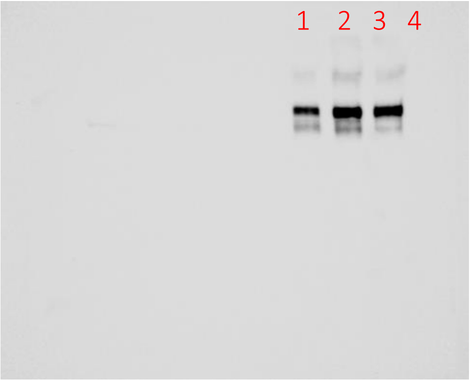

Sample:
1: hTM control
2: hTM TGFß
3: hTM DEX
4: Precision Plus Protein Standards All Blue

C

Westernblot FN1 mTM (Stain Free Gel)

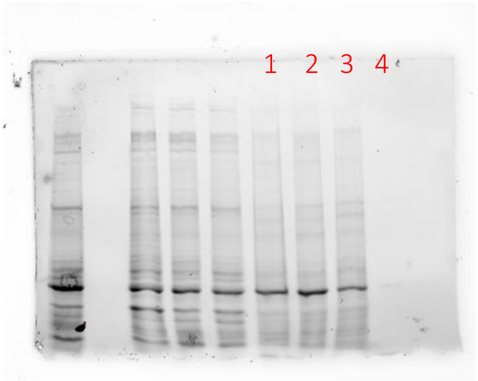

Westernblot FN1 mTM (Stain Free Blot)

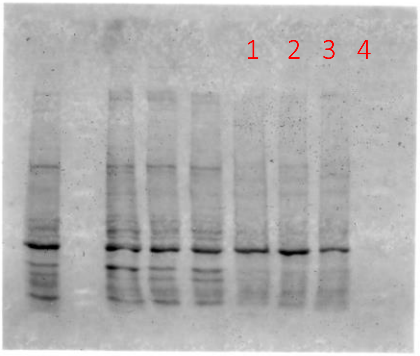

Westernblot FN1 mTM (Cy5)

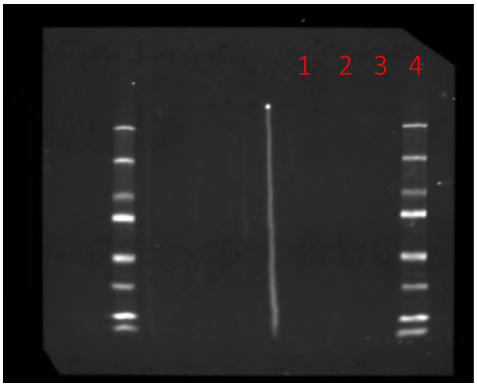

250 kD
150 kD
100 kD
75 kD
50 kD
37 kD
25 kD
15 kD

Westernblot FN1 mTM (Chemiluminescence)

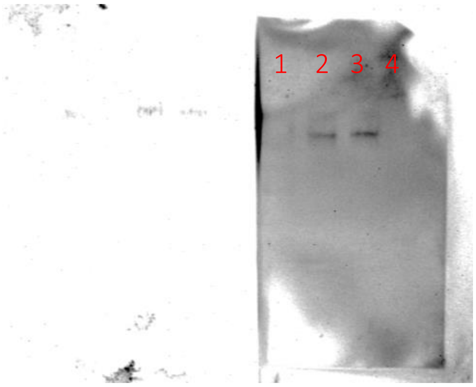

Sample:
1: mTM control
2: mTM TGFß
3: mTM DEX
4: Precision Plus Protein Standards All Blue

D

Fig. 2 C+D

Fluorbeads (FB) hTM	Numbers	Percentages
FB-positive cells	5339	64.99%
FB-negative cells	2867	34.90%
Total	8215	

Fluorbeads (FB) mTM	Numbers	Percentages
FB-positive cells	1393	71.58%
FB-negative cells	553	28.42%
Total	1946	

Fig. 4 B+C

CLANS hTM	Mean	SD	N
Control	12.73%	6.33%	10
TGFB	63.89%	8.06%	12
DEX	48.66%	9.03%	15

CLANS mTM	Mean	SD	N
Control	13.27%	8.55%	14
TGFB	66.37%	8.09%	12
DEX	46.49%	7.89%	15

Fig.5

ACTA2 hTM	Mean	SD	N
Cont	1	0	3
TGFß	2.20	0.82	3
Dex	2.14	0.45	3

ACTA2 mTM	Mean	SD	N
Cont.	1	0	3
TGFß	2.38	1.367	3
Dex	0.72	0.479	3

Fig.6

FN1 hTM	Mean	SD	N
Cont	1	0	3
TGFß	1.88	0.48	3
Dex	1.06	0.47	3

FN1 mTM	Mean	SD	N
Cont	1	0	3
TGFß	8.89	6.01	3
Dex	11.83	9.08	3
